# Parents’ acceptance of human papilloma virus vaccination for their daughters in adet town, North Gojjam zone, Northwest Ethiopia: A mixed method study

**DOI:** 10.1371/journal.pone.0330911

**Published:** 2025-08-26

**Authors:** Bezawit kassa, Asiya Mohammed, Gizachew Tadesse Wassie

**Affiliations:** 1 Department of Epidemiology and Biostatistics, School of Public Health, College of Medicine and Health Sciences, Bahir Dar University, Bahir Dar, Ethiopia; 2 Department of Epidemiology and Biostatistics, School of Public Health, College of Medicine and Health Sciences, Bahir Dar University, Bahir Dar, Ethiopia; 3 Department of Epidemiology and Biostatistics, School of Public Health, College of Medicine and Health Sciences, Bahir Dar University, Bahir Dar, Ethiopia; IAVI, UNITED STATES OF AMERICA

## Abstract

Human papilloma virus vaccination is an effective way to reduce cervical cancer. Although the health of adolescents is the priority goal across the globe, including Ethiopia, parent s’ acceptance of Human papilloma virus vaccination for their daughters becomes a big challenge in Ethiopia. This study aimed to assess parent’s acceptance of human papilloma virus vaccination for their daughters and associated factors in Adet town, northwest Ethiopia, 2024. Community based mixed method study was employed from May 24 to June 27, 2024. For quantitative data, the sample size was 319 and systematic random sampling technique was used to select study participants. Structured questionnaires were administered for quantitative data, and interview guides were used for qualitative data. The quantitative data was coded, entered and cleaned using Epi info software and exported to SPSS version 25 for analysis. Descriptive statistics, bivariable and multivariable logistic regression analysis were used. Variables with p**-**value of <0.05 with 95% confidence interval and adjusted odds ratio in multivariable analysis were considered as statistically significant factor for the outcome variable. Thematic analysis approached was employed to analyse qualitative data. Results were presented in tables, texts, charts and graphs. In this study, 249(78.1%) with 95%CI: 73.0–82.4) of parents accept HPV vaccination for their daughters. Knowledge (AOR = 2.96, 95% CI: 1.43–6.10), attitude (AOR = 3.47, 95%CI: 1.71–7.04), subjective norms (AOR = 3.20, 95% CI: 1.56–6.51) and safety concern (AOR = 8.20, 95%CI: 3.45–19.49) were significantly associated factors with parents’ acceptance of HPV vaccine. Qualitative results identified barriers to parental acceptance of the HPV vaccine for daughters, including fear of side effects like infertility, perceiving it as contraceptive method, lack of HPV knowledge, and absence of institutional accountability. Facilitators included influential stakeholder engagement, positive perceptions of the vaccine’s benefits, and confidence in its safety and efficacy. The result of this study seems promising as a more than two thirds of women accept to vaccinate their daughters against HPV though there are still misconceptions, safety and efficacy concerns. To foster cervical cancer prevention efforts, parents’ health education should address vaccination safety concerns, improve HPV knowledge, and foster positive attitudes towards vaccinating daughters.

## Introduction

Cervical cancer, the second leading cause of cancer-related mortality and morbidity among women globally after breast cancer, poses a significant threat to women’s health, including adolescents [1]. Over 70% of cervical cancer cases are attributed to persistent infection with high-risk human papillomavirus (HPV) types 16 and 18 [1–6]. HPV is highly contagious virus and transmitted sexually, which is major public health concern both nationally and internationally [7,8]. This characteristic renders it a silent threat among sexually active adolescents, with over 75% of sexually active individuals estimated to acquire HPV during their lifetime [3,9–11].

Primary prevention through HPV vaccine has the capacity in reducing cervical cancer incidence globally by 70% [7]. Since 2006, two vaccines the bivalent named (Cervarix), which immunizes against strain16 and 18 and the quadrivalent called (Gardasil),which gives additional immunity against two genital wart strains, HPV 6 and 11 have been widely implemented [9,11–13]. Both vaccines are effective and safe administered intramuscularly. The vaccine is given for adolescents before they become sexually active to increase its effectiveness [6,9]. Gardasil 9 is the latest version of HPV vaccine introduced in 2016 [14]. According to World Health Organization (WHO) recommendation, two doses of HPV vaccination to adolescent females aged 9–14 years is administered [3,13]. In Ethiopia, HPV vaccine was introduced in 2018 with the aid of global alliance for vaccination and immunization (GAVI), which is administered to adolescent females aged 9–14 years through school based approach [3,15,16].

The WHO’s global strategy to eliminate cervical cancer as a public health threat by 2030 aims for 90% HPV vaccination coverage among 15-year-old girls [17,18]. Modeling studies estimate that high vaccine uptake could prevent over 2.1 million deaths [19]. For vaccinations to be effective, high uptake is mandatory. In addition, with a 50% and above HPV vaccination coverage, HPV infections should decrease by 68% through herd immunity and cross-protection [9]. However, vaccine hesitancy and parental refusal remain critical barriers to achieving these targets [7]. Parental acceptance is particularly crucial; as vaccination programs for adolescents aged 9–14 years often require caregiver consent [7,20,21].

Global disparities in HPV vaccine acceptance persist, driven by sociocultural and geographical factors [4,5,10]. Colombia was the first country to implement the HPV vaccine, achieving uptake rates of 97.5% for the first dose and 96.7% for the second in 2012. However, hesitancy later reduced uptake by 14% [22–25]. Similarly, Saudi Arabia (94%) [8], Poland (43.1%) [26], Denmark, Australia, and Japan report significant parental reluctance.

In Africa, vaccine acceptance is low; in South Africa, uptake decreased by 21.4% and 26% between 2014 and 2016, with only 32.2% of adolescent girls vaccinated [9], majority of the respondents (91.2%) did not accept HPV vaccination for their daughters [28]. In Nigeria, vaccine acceptance remains poor despite high cervical cancer rates [27]. In Tanzania, only 6.9% vaccinated their daughters [6], and in Zambia, many eligible girls were not vaccinated due to parental non-consent [18]. Similarly, a study in Kenya found that 30% of parents were unwilling to accept HPV vaccination for their daughters [13].

Ethiopia faces similar challenges. Studies in Jimma town reporting 39.02% hesitancy [29], and in Ambo found that HPV vaccine awareness among parents was as low as 20%, impacting acceptance [30]. In Debremarkos, many parents opposed vaccination for various reasons [31], while a study in Debre Tabor showed only 44.8% were willing to vaccinate their daughters [22], and over half of participants in Addis Zemen expressed fear of side effects, and a lack of trust in the vaccine’s safety contributed to refusal [32].

Adolescent health remains a public health priority in Ethiopia and globally. However, parental misconceptions about cervical cancer etiology, prevention, and vaccine safety hinder HPV vaccine acceptance, particularly in resource-limited settings [24,33].

Despite these facts, HPV vaccination uptake among adolescent girls remains limited, mainly parents misconception about the cause and prevention of cervical cancer and poor acceptability of HPV vaccination, which becomes an obstacle in developing countries including Ethiopia [2,9,15,34–36]. Addressing these barriers requires community-level studies to assess parental attitudes and inform targeted interventions [2,9]. Despite this need, research on parental acceptance of HPV vaccination in Ethiopia remains scarce [16]. Therefore, the current study aimed to assess parents’ acceptance of human papilloma virus vaccination for their daughters and associated factors using mixed method study in Adet town, northwest Ethiopia.

## Materials and methods

### Study area and period

The study was conducted in Adet town, North Gojjam Zone, Northwest Ethiopia from May 24 to June 27, 2024. Adet town is found 43 km due East from Bahir Dar, the capital city of Amhara Region. The town has five urban kebeles (the smallest administrative unit in the country) with a total population of 59,470 as of 2023. Based on the health profile of Adet town administrative health office, the town has one health center with five health posts, one primary government hospital and five private primary clinics, four private medium clinics, one pharmacy and five drug stores which deliver routine preventive and curative health services to the community. The town has seven primary schools, two secondary and one preparatory school which deliver routine service for the community.

### Study design

Community based Cross-sectional concurrent mixed method was used to assess parents’ acceptance of human papilloma virus vaccination for their daughters and associated factors in Adet town, north Gojjam zone, northwest Ethiopia.

### Study participants

All parents who have daughters from 9–14 years old living in Adet town was source population. Sample participants for the quantitative study were drawn from this population using systematic sampling technique. For qualitative part, study participants were purposively selected parents with different backgrounds, and other stakeholders.

### Sample size and sampling procedure

For quantitative part sample size was determined by using double population formula using Epi-Info TM 7 software considering the following statistical assumptions: 95%CI, power 80%, 1:1 exposed to unexposed ratio and the corresponding OR(2.2) of significantly associated predictor variables from previous similar study [2,22,32], using wealth status, the sample size was 290. Then by adding 10% non-response rate, the final sample size was 319.

The sample size for the qualitative part of the study was determined by the information saturation criteria. A total of 13 (12 in-depth interview (IDIs) and (two key informant interview (KIIs) participants with different background participated in the study. In-Depth Interviews were conducted with parents, while KIIs were conducted with District Expanded Program of Immunization officers and Health Extension Workers. Participants were selected based on their engagement in decision making, expertise and knowledge of community health practices, vaccination programs, and healthcare access, with the aim of gathering insights on community perceptions, barriers to healthcare, and policy implementation.

### Measurements

The main outcome of this study was Parents’ acceptance of HPV vaccination for their daughters assessed with six yes or no questions. Parents scoring at or above the mean on the acceptance assessment were considered to accept HPV vaccination (Yes), while those scoring below the mean were considered not to accept it [4,22].

Socio-demographic variables (sex, age, relationship status, religion, educational status, occupation, average monthly income, marital status, and media exposure), reproductive and other health-related factors (number of daughters and family history of cervical cancer), and parental behavior-related factors (knowledge, perceived behavior, subjective norm, safety concerns, and attitudes towards HPV infection and vaccination) were assessed to determine whether they had an association with the outcome variable or not.

Knowledge level of participants was assessed with seven yes or no questions. Parents who scores at the mean and above were considered as having good knowledge, if not poor knowledge [3,4,16]. Attitude was measured using a five-point Likert scale; from strongly disagree to strongly agree. Those who scored the mean and above were considered to have a positive attitude, while those who scored below were considered as having a negative attitude [3,4].

Subjective norms, defined as the perceived expectations of significant others regarding the behavior, can significantly influence societal acceptance or rejection of vaccination. Subjective norms were measured using a five-point Likert scale (strongly agree to strongly disagree), with scores at or above the mean considered positive, and scores below the mean considered negative [4].

Perceived behavioral control, which is the capability to perform a behavior, the participant’s self-rule and intention of difficulty or ease to take HPV vaccination. It was measured with five Likert scale from strongly agree to strongly disagree and those who score the mean above have positive perceived behavior, otherwise negative perceived behavior [4].

Safety Concern about HPV vaccine was also assessed with five Likert scale from strongly disagrees to strongly agree. Participants who scores the mean and above were considered as having high safety concern otherwise low safety concern [37].

### Data collection tools and procedures

Quantitative data were collected using structured face to face interviewer-administered questionnaires adapted from different literatures [2–4]. The questionnaires were translated from English to Amharic and back-translated for consistency. Three diploma nurses and one BSc nurse, trained by the principal investigator, collected the data, with daily checks for completeness.

For the qualitative part, face-to-face IDI and KIIs were conducted using an open-ended interview guide. The guide explored barriers and facilitators to parents’ acceptability of HPV vaccination of their daughters. The interviewer introduced the purpose of the study to the participants and assured them of confidentiality before obtaining the informed verbal consent. All the interviews were audio recorded with permission from the participants and conducted in private, accessible locations.

### Data quality control

Two days training was given for data collectors and supervisors. The internal reliability of the questionnaires was checked by Cronbach’s α, which were acceptable,0.797, 0.809, 0.776, 0.745 and 0.792 for Safety concern, perceived behavior, Subjective norm, Attitude and Knowledge respectively. Pretest was conducted on 5% of the sample size prior to the actual data collection in other study area (Debremawi district), to check consistency and clarity of the questionnaires. The result of the pretest was used to make modification for the questionnaires. The investigator and supervisor supervised and reviewed every data collection procedure and completeness of the questionnaires, logical consistency and correction was made. The principal investigator collected the completed questionnaires daily and was responsible for the coordination and on spot supervision of overall data collection process.

### Rigor of the study

To ensure rigor, the qualitative data collection guides were initially developed in English and then translated into Amharic (local language) and back. Interviews were transcribed verbatim, translated to English, and field notes were added for clarification. English transcripts underwent multiple reviews for coding, theme development, and sub-theme organization using MS Word. Trustworthiness was ensured by meeting criteria for credibility, transferability, dependability, and conformability.

### Data processing and analysis

Data were checked, coded and entered into epi-info and exported to SPSS version25 for analysis. Descriptive statistics were used to show the characteristics of the study participants. Binary logistic regression model was fitted to identify statistically significant explanatory variables. Those variables with p-value of <0.25 were entered to multivariable logistic regression model to identify possible confounding variables. The adjusted odds ratio (AOR) with 95% CI and p-value of<0.05 was used to determine statistically significant association. Hosmer-Lemeshow goodness of fit test was checked and it was well fitted (0.871). Results were presented in texts, tables, charts and graphs.

The qualitative data was analyzed through thematic approach. Major and subthemes were created from the text itself through repeated reading. After reading the transcripts, the investigator identified emergent themes and then coded each theme to delineate individual topics. Statements were grouped by code to the corresponding themes. The findings were presented in narratives by thematic areas.

### Ethics approval and consent to participate

Ethical approval was obtained from the ethical review board of Bahir Dar University, College of Medicine and Health Sciences at 13/9/2016 and RDD/1977/2016 with protocol number 931/2024. At all levels letter of support was given to the relevant administrative officials. Written informed consent was obtained from all participants after explaining the study’s objective. Participants were informed of their right to participate, withdraw at any time, and ask questions. All data were kept private and confidential, with names and addresses excluded from records. Only the investigator had access to the collected data.

## Results

### Socio-demographic characteristics of participants

In this study, a total of 319 study participants were included with a response rate of 100%. The mean age of the participants’ was (44.67, SD ± 9.09565) year and (67.4%) were above 40 year. Almost half (51.1%) of them were females. Most of the participants (91.8%) were Christian by religion and (90.6%) of participants were married. Regarding education and occupation, (44.8%) of the participants were completed college and above (38.6%) of the participants were government employees. More than half (55.2%) had average monthly income above 4000 ETB. Three fourth (87.8%) of participants were exposed to media (Table 1).

**Table 1:** Socio-demographic related characteristics of study participants in Adet town, Northwest Ethiopia, 2024, (n = 319).

Variables	Category	Frequency	Percent(%)
Age in year	<=29	9	2.8
	30-39	95	29.8
	>=40	215	67.4
Sex	Male	156	48.9
	Female	163	51.1
Religion	Christian	293	91.8
	Muslim	26	8.2
Relationship	Father	153	48
	Mother	158	49.5
	Guardian/caregiver	8	2.5
Current marital status	Single	30	9.4
	Married	289	90.6
Educational status	Unable to read and write	34	10.7
	Able to read and write	60	18.8
	Primary education	29	9.1
	Secondary education	53	16.6
	College and above	143	44.8
Monthly income	Less than 2000ETB	37	11.6
	2000-4000ETB	106	33.2
	Above 4000ETB	176	55.2
Occupation	Housewife	61	19.1
	Merchant	94	29.5
	Government employed	123	38.6
	Self-employed	41	12.9
Media exposure	Unexposed	39	12.2
	Exposed	280	87.8

### Knowledge about HPV infection and vaccination

The mean score of the study participants’ knowledge was (10.47, SD ± 2.22593) and majority of them 169 (53%) had good knowledge, while 150 (47%) had poor knowledge (Table 2).

**Table 2:** knowledge of parents to accept HPV vaccination for their daughters in Adet town, northwest Ethiopia, 2024, (n = 319).

Variables	Responses	Frequency	Percent (%)
Have you ever heard about cervical cancer?	Yes No	248 71	77.7 22.3
Do you know the risk factors of cervical cancer?	Yes No	153 166	48.0 52.0
Have you ever heard of Human Papilloma virus?	Yes No	141 178	44.2 55.8
Do you think HPV can be transmitted through sexual contact?	Yes No	172 147	53.9 46.1
People can transmit HPV to their partner Even if they have no symptoms of infection.	Yes No	139 180	43.6 56.4
Do you think HPV can heal by itself without any treatment?	Yes No	78 241	24.5 75.5
Do you know cervical cancer has a vaccine?	Yes No	192 127	60.2 39.8

### Attitude, subjective norm, perceived behavior and safety concern of parents about HPV vaccine

The attitude mean score of the study participants was (21.88, SD ± 6.00627), and the majority of the study participants 185 (58%) had positive attitude towards HPV vaccine. The subjective norm mean score of the participants was (14.19, SD ± 3.78592), majority of the study participants 199(62.4%) had positive subjective norm. Half of the respondent’s 162(50%) were agreed that professional counseling was influential for parents acceptance of HPV vaccinations for their daughters. The perceived behavior mean score of the participants was (15.14, SD ± 3.391). The majority of the participants, 173 (54.2%) had positive perceived behavior. Besides, nearly half of the participants 165(51.7%) agreed that the vaccination of their daughter against HPV was possible. The mean score of the participants’ safety concern was (9.75, SD ± 3.54083). Majority of them 176 (55.2%) had low safety concern for HPV vaccine (Table 3).

**Table 3:** Attitude, subjective norm, perceived behavior and safety concern about HPV vaccine of parents to accept HPV vaccine for their daughters in Adet town, northwest Ethiopia, 2024,(n = 319).

Attitude					
Variables	Strongly Dis Agree (N %)	Disagree (N %)	Neutral (N %)	Agree (N %)	Strongly Agree(N%)
There is a risk for young women to contract HPV infection	28(8.8)	71 (22.3)	27 (8.5)	143(44.8)	50(15.7)
My daughter/s may have cervical cancer in the future	52 (16.3)	100(31.3)	60(18.8)	96(30.1)	11(3.4)
Cervical cancer is a severe disease	14 (4.4)	32 (10.0)	72(22.6)	60(18.8)	140(43.9)
Cervical cancer causing virus(HPV) vaccines can effectively prevent cervical cancer	40(12.5)	79 (24.8)	50(15.7)	110(34.5)	40(12.5)
I am afraid of the side effects of HPV vaccination to my daughter/s	67 (21)	112 (35.1)	57(17.9)	79(24.8)	4(1.3)
I consider HPV vaccine is harmful	107 (33.5)	131 (41.1)	43(13.5)	35(11)	3(0.9)
I am afraid of being HPV vaccination cause my daughter to be sexually active early	127 (39.8)	117 (36.7)	50(15.7)	23(7.2)	2(0.6)
Girls who have the HPV vaccination might be more likely to have unprotected sex	94 (29.5)	110 (34.5)	60(18.8)	48(15.0)	7(2.2)
**Subjective norm of participants**					
Health professional counseling to vaccinate for HPV is influential	17 (5.3)	15 (4.7)	16(5.0)	162(50.0)	109 (34.2)
Teacher’s or principal’s recommendation to vaccinate for HPV is influential	44 (13.8)	48 (15)	30 (9.4)	151(47.3)	46 (14.4)
Recommendations of friends or families to vaccinate for HPV are influential	41 (12.9)	45 (14.1)	38(11.9)	148(46.4)	47(14.7)
Spiritual leader’s recommendation to vaccinate for HPV is influential	49(15.4)	16(5.0)	61(19.1)	125(39.2)	68(21.3)
**Perceived behavior of participants**					
For me, the vaccination of my child against HPV is possible	8(2.5)	34(10.7)	15(4.7)	165(51.7)	97(30.4)
If I wanted to vaccinate my daughters against HPV, it would be easy	8(2.5)	45(14.1)	27(8.5)	157(49.2)	82(25.7)
It depends entirely on me to vaccinate my child against HPV	17(5.3)	56(17.6)	52(16.3)	133(41.7)	61(19.1)
I feel control on vaccinating of my child against HPV	7(2.2)	45(14.1)	29(9.1)	149(46.7)	89(27.9)
**Safety concern about HPV vaccine**					
I feel that giving my daughter HPV vaccine could be like performing an experiment on her.	85(26.6)	138(43.3)	27(8.5)	41(12.9)	28(8.8)
I feel that HPV vaccine may cause problems getting pregnant in the future	62(19.4)	160(50.2)	53(16.6)	35(11.0%)	9(2.8)
I am afraid of vaccination of my daughters	66(20.7)	170(53.3)	25(7.8)	43(13.5)	15(4.7)
I am concerned about HPV vaccination side effects.	40(12.5)	98(30.7)	69(21.6)	91(28.5)	21(6.6)

### Parents’ acceptance of HPV vaccine for their daughters

In this study, 78.1% (95%CI:73.0–82.4) of parents accept HPV vaccination for their daughters. It was assessed with six yes or no questions. Parents scoring at or above the mean on the acceptance assessment were considered to accept HPV vaccination (Yes), while those scoring below the mean were considered not to accept it. More than two third (90.3%) of the participants believe that HPV vaccine was important for health and 85.6% of parents were willing to vaccinate their daughters against HPV infection if it is safe. The majority (88.4%) of the participants claim that they will make effort to have their daughters to get HPV vaccination. About two third (75.5%) of the participants also claim that they will regret if their daughter does not get HPV vaccine. The majority (75.9%) of the participants also think that daughters should get HPV vaccine before they start sexual contact. In addition, 78.4% of the participants recommended HPV vaccine for others (Table 4).

**Table 4:** Parents acceptance of HPV vaccination for their daughters in Adet town, northwest Ethiopia, 2024, (N = 319).

Variables	Responses	Frequency	Percent
Are you willing to vaccinate HPV vaccination for your daughter that can protect against HPV infection?	Yes	273	85.6
	No	48	14.4
For me, it is an important item of health management to have my daughters get the HPV vaccination	Yes	288	90.3
	No	31	9.7
I will make efforts to have my daughters get the HPV vaccination	Yes	282	88.4
	No	37	11.6
I will regret if my daughters cannot get HPV vaccination	Yes	241	75.5
	No	78	24.5
Daughters should get HPV vaccine before sexual contact	Yes	242	75.9
	No	77	24.1
I recommend HPV vaccine to others	Yes	250	78.4
	No	69	21.6

**Fig 1:**
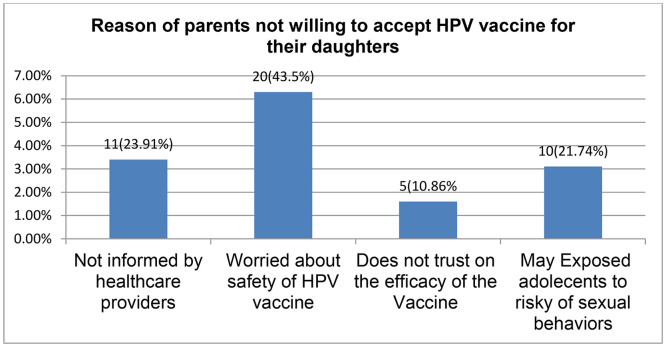
Reasons cited by parents for unwillingness to accept HPV vaccination for their daughters.

The main reasons of those parents unwillingness to accept HPV vaccination for their daughters were their worries about safety of the vaccine, not informed by health professionals and may expose adolescent’s for risky sexual behaviors (Fig 1).

### Barriers and facilitators of parents’ HPV vaccine acceptance for their daughters

From the qualitative study, four main themes and eight subthemes were identified. The major themes include perceptions and knowledge about of HPV infection, sources of information and the importance of the HPV vaccine, fears regarding side effects and beliefs about the HPV vaccine, influential stakeholders, and a lack of responsibility.

### Theme 1: perceptions and knowledge about of HPV infection

#### Subtheme 1.1: perception of HPV infection.

Most participants perceived that early sexual intercourse, early marriage, and unsafe sexual practices (such as rape) could lead to HPV infection. One participant stated:

*“If childhood marriage or early sexual intercourse occurs, or if sexual intercourse during childhood happens for an extended period, cervical cancer can be caused by these factors.” (Participant 7, age 38, female).*

Another participant connected unsafe sex and rape to cervical cancer, stating:

*“We don’t know what diseases men are infected with or what illnesses they may have. When daughters are raped, this causes bleeding, which can lead to various illnesses, including cervical cancer.” (Participant 9, age 42, female).*

#### Subtheme 1.2: poor knowledge about the causes of cervical cancer.

Many participants demonstrated a lack of understanding regarding the risk factors associated with HPV infection. They believed that exposure to sunlight while urinating and poor hygiene was significant risk factors. One participant articulated this belief:

*“We believe that the risk factor for cervical cancer is a lack of hygiene. If you don’t clean yourself, something harmful and dirty can enter the body and cause this disease.” (Participant 10, age 36, female).*

Another participant expressed similar concerns:

*“Especially when we urinate on hot soil that has been exposed to the sun, it leads to cervical cancer. When we urinate, the soil becomes wet and rises like steam; this is what exposes us to this disease.” (Participant 4, age 39, female).*

A male participant further elaborated on this belief:

*“When you urinate on hot or sunny ground, steam rises. I am particularly worried that this may cause cervical cancer. When women urinate in the field, it heats up, and when the urine meets the hot ground, it vaporizes and can enter the uterus. The hot ground creates steam, and this is my main concern. Yes, it enters the uterus; we refer to this as (MECH).” (Participant 3, age 40, male).*

A key informant noted that the community’s knowledge on this issue remains limited:

*“Many societal views are still not fully understood, and the first priority for improving these perspectives is to create a better understanding of health promotion and the benefits of vaccination. The knowledge is indeed partial.” (KI -1, age 40, male).*

### Theme 2: Source of information and perceived benefit of HPV vaccine

This theme also contains two subthemes.

#### Subtheme 2.1: Source of information about HPV vaccine.

In these subthemes majority of the respondent’s expressed their source of information about HPV vaccination were health professionals and TV even though there were parents who were never heard about HPV vaccine. One of the respondents expressed this as follows;

“*Yes...we have information, although not complete, because we hear from health professionals sometimes and through advertisements on television and radio” (participant6, age 38, male).*

On the other hand*,* the other respondent expressed as follows;

*“I get information from a doctor and my daughters also take the vaccination, because of the advice what I got from the medical professionals.”(participant 9, age 42, female).*

#### Subtheme 2.2: Perceived benefit of HPV vaccine.

In this subtheme, the respondent’s expressed that HPV vaccine is used to prevent against cervical cancer and it was used to become healthy. One of the study participants expressed that;

*“This cervical cancer vaccine is about prevention. The vaccine is about prevention.* *If we prevent Pre-cancer, it means that we prevent cancer.” (Participant 7, age 38, female).*

In addition another respondent’s also expressed the same thing*; “The reason why I believe the importance of vaccination is that a person should be vaccinated early. It is prevention, right? If the vaccine is early, it will prevent the children or women from getting the disease. It is very important because it protects from cervical cancer.” (Participant 9, age 42, female).*

### Theme 3: fear of side effects and Belief about HPV vaccine

In this theme, the participants were expressed their misconception about HPV vaccine side effects, to the reverse, most of the participants had good belief about HPV vaccine.

#### Subtheme 3.1: Fear of side effect about HPV vaccine.

In this subtheme, most of the participants were expressed their fear of side effects or concern about HPV vaccinations for their daughters as follows;

“*It’s just sexual abuse, because we think the vaccine as pregnancy preventive method...Just to sum up it, we believe that the vaccine is a protection against pregnancy.”(participant2,age 32, female).*

The other respondent also expressed that HPV vaccine leads to infertility and they consider this as method of birth control.

*“I am especially afraid of two things, birth control... because now you know what this is that scares me. When birth control is given only to women, it is women who take a lot of time and get injected. I’m a little disappointed with that and I don’t like to pass away without being replaced and I am afraid that my children will be infertile.”* (*Participant 4, age 39, female).*

Overall, one of the KIs also expressed the same thing when we asked about is there any rumor among the community towards HPV vaccination of their daughters. This suggests that the spread of misinformation, particularly linking the vaccine to family planning, can negatively impact its acceptance.

*“spreading false rumors about why my daughter was injected, for example...family planning, which I didn’t know before was injected, our children are about to suffer...one of these kinds of things is to emphasize the false results; leave the best and highlight the bad and it happens”.(KI-1,age 40,male).*

#### Subtheme 3.2: Belief about HPV vaccine.

In this subtheme, most of the participants expressed their belief about HPV vaccine can prevent cervical cancer. One participant expressed this as follows:

*“I think it would be better if we vaccinate the daughters on time and protect children from cervical cancer. You can’t get the vaccine financially even now we know the current situation. I don’t think there is anyone who has such a bad attitude; I think it is good starting from me.” (Participant 8, age 43, male).*

“*It is important for a person to prevent the disease before contracting it. I believe that it is very important that this cervical cancer vaccine is given in order to easily solve the problem of our sisters, our children and our mothers.”(Participant 11, Age, 32.male).*

### Theme 4: Involvement of influential stakeholders and lack of responsibility

This theme expressed the following sub themes.

#### Subtheme 4.1: Influential stakeholder’s involvement.

In this subtheme, participants expressed that counseling of influential stakeholders was important to change community’s behavior towards parent’s acceptance of HPV vaccinations for their daughters. One of the study participants expressed like this.

*“The starting point for all of this is counseling and after a person trusts it then they come close to that, after that their consent should be asked and the vaccination should be given. Above all, professional counseling is needed” (participant 6, age 38, male).*

Another participant also expressed this thing:

*“Yes, counseling services should be given in a broad sense, that is, first of all by the religious leaders, if they give an explanation for people available at the church and give suggestions for us, we will have more knowledge, then the society will accept the vaccine.” (Participant 2 age 32, female).*

One of the KIs also expressed the same thing.

*“When a religious father tells them about HPV vaccine for parents who don’t have a good attitude towards the vaccine, they think that it’s good if counseling comes from religious leaders. Yes, they listen them very much rather than health professionals. If we work together with them, there will be community’s behavioral change towards HPV vaccine. Yes, there is no question” (KI-2 age 35, female).*

Similarly, the other key informant expressed as follows:

*“Different stakeholders will bring a better change for parents’ acceptance of HPV vaccine. The combination of each...the influencer, especially now when we see it differently, or when I see it as an officer, especially the religious leaders have a voice.” (KI- 1, age 40, male).*

#### Subtheme 4.2: lack of responsibility.

In this subtheme KIs expressed their fear of HPV vaccine shortage.

*“The more we went, the more it became scarce. So from the top, the federal government wanted us to plan for the 9-14 year olds; it means that the supply is limited. That is why it came from the top down to prevent them from reaching the stage where they are expected to reach the age of sex before they pass”(KI-1,age 40,male).*

The other key informant also described as follows.

*“The existing supply is still good according to our perception, but it has some problems.The sustainability of vaccine access is not certain. Now it is thought that there was a shortage of vaccines, so let’s start from 14 years old, it will go down. The vaccine was given from 9-14” (KI-2, age 35 female).*

### Factors associated with parent’s acceptance of HPV vaccination for their daughters

In the bivariable analysis, educational status, current marital status, knowledge,attitude, perceived behavior, subjective norm, safety concern about HPV vaccine and media exposure were candidates for multivariable analysis at p- value <0.25.

In the multivariable analysis, knowledge, attitude, subjective norm and safety concern about HPV vaccine were significantly associated with the outcome variable at p-value <0.05. Parents who had good knowledge about HPV infection and HPV vaccination were 2.96 times more likely to accept HPV vaccination for their daughters compared with parents with poor knowledge (AOR = 2.96, 95%CI,(1.43–6.10).

Besides, parents who had positive attitude towards HPV vaccination were 3.47 times more likely to accept HPV vaccination for their daughters compared with parents who had negative attitude towards HPV vaccination (AOR = 3.47,95%CI, (1.71–7.04). Furthermore, parents who had positive subjective norm were 3.19 times more likely to accept HPV vaccination for their daughters when compared with parents who have negative subjective norms (AOR = 3.19, 95% CI (1.56–6.51). Moreover, parents who had low safety concern about HPV vaccination were 8.20 times more likely to accept HPV vaccination for their daughters compared with parents who had high safety concern about HPV vaccination (AOR = 8.20, 95%CI (3.45–19.49)(Table 5).

**Table 5:** Binary and multiple logistic regression analysis of factors associated with parents acceptance of HPV vaccinations for their daughters in Adet town, northwest Ethiopia, 2024, (n = 319).

Variables	Categories	Acceptance		COR(95%CI)	AOR(95%CI)	p-value
		Not Accept	Accept			
Relationship	Father	33	120	1	1	
	Mother	33	125	1. 04 (0.61-1.80)	1.00(0.50-2.00)	0.997
	Caregiver	4	4	0.28(0.07-1.16)	0.39(0.06-2.54)	0.322
Educational status	Unable to read and write	10	24	1	1	
	Able to read and write	14	46	1.37(0.53 −3.54)	1.452(.442-4.769)	0.539
	Primary education	11	18	0.68 (0.24-1.95)	0.46 (0.12-1.87)	0.279
	Secondary education	11	42	1.59 (0.59 −4.29)	0.74(0.20-2.88)	0.661
	College and above	24	119	2.07(0.88-4.87)	1.58 (0.51 −4.88)	0.424
Current marital status	Single	4	26	1	1	
	Married	66	223	0.52 (0.18-1.54)	0.44 (0.12-1.68)	0.230
Attitude	Negative	50	84	1	**1**	
	Positive	20	165	4.91 (2.75-8.78)	3.47(1.71-7.04)*	**0.001**
Subjective norm	Negative	42	78	1	**1**	**0.001**
	Positive	28	171	3.29(1.90 −5.69)	3.19(1.56 −6.51)*	
Perceived behavior	Negative Positive	48 22	98 151	1 3.36 (1.91 −5.92)	1 1.37 (0.63 −2.98)	0.426
Safety concern	High	59	84	1	**1**	**<0.001**
	Low	11	165	10.54(5.26-21.12)	8.20 (3.45 −19.49)**	
Knowledge	Poor	49	101	1	**1**	**0.003**
	Good	21	148	3.42(1.93 −6.05)	2.957(1.433-6.101)*	
Media exposure	Unexposed	5	34	1	1	0.148
	Exposed	65	215	0.49(0.18 −1.30)	0.42(0.13-1.37)	

## Discussion

In this study, 78.1% of parents accept HPV vaccinations for their daughters. This finding was in line with other studies done in Thailand (77%), India (78%) and London (75%) [10,19,36]. This finding was also in line with a study done in Western Nigeria(79.2%) [7], which might be similarity in some of Socio-demographic characteristics of the study participant in which the mean age of the respondent’s and level of education were almost similar with the current study in Western Nigeria. This study was also consistent with other studies done in different parts of Ethiopia, Bench Shako (79.5%), Gondar (81.3%), Debre Tabore (79.1%), Addis Zemen (80.3%) [2,4,32,38].

However, the current finding was higher than previous findings from Turkey (45.5%), United States (48%) and Saudi Arabia, Jeddah where 94% of parents were unwilling to accept HPV vaccine for their daughters [8,20,37]. The reason for this discrepancy could be in Jeddah, there was online survey and the study was institution based where only parents attending hospital were involved in contrast to the current finding. This finding was also higher than the study done in Nigeria, Abuja (62.8%), Zambia, Lusaka (53.8%) and Western Kenya (70%) [13,18,27]. This discrepancy could be the difference in study period and 92% of the study participants had poor knowledge of HPV vaccine and the participants were only females attending antenatal care in Nigeria. This difference might also be due to non-availability of HPV vaccine (64.7%) and high cost of HPV vaccine (60.8%) in Kenya in contrast to the current study where HPV vaccine was free and available in the study setting. The current finding was also higher than the study done in Debre Taboure (44.8%) [22] and study done in Woldia, Ethiopia (72.9%) [39].

This finding was lower than the studies done in Poland (85.1%), Indonesia (91%) and China in different periods (87.6% and 83.3%) [21,40,41], this difference could be the difference in study settings and method of data collection, where a study in Poland shows that the data was collected in parents and teachers meeting, which might be affected by selection of the study participants. This finding was also lower than the study done in Nigeria, Lagos (88.9%) [6], this difference could be routine immunization coverage was high and 97.6% of immunization was compulsory in Lagos. In addition, the study in Nigeria considers only mothers and doesn’t consider fathers in contrast to the current study.

The current finding was also lower than the study done in Hadiya (84.9%), Addis Ababa, and Ethiopia (94.3%) [3,16]. This difference could be due to the difference in study setting and participant’s level of knowledge and Attitude towards HPV vaccinations, where high level of literacy and access to better health care service about HPV vaccine to the community was expected to be high in Addis Ababa.

This finding revealed that knowledge on HPV vaccine and HPV infection was significantly associated with parent’s acceptance of HPV vaccinations for their daughters. This finding was supported by other similar studies done in Poland [26], Kenya [13], Western Nigeria [7] and in Ethiopia, Gondar, Debre Tabore and Woldia in different periods [2,4,22,39]. The reason for this could be parents being aware of the severity of cervical cancer and they know that this disease was the main cause of maternal death.

In addition, this finding also revealed that parents who have positive attitudes about HPV vaccine have significantly associated with parent’s acceptance of HPV vaccinations for their daughters. This finding was also supported by other similar studies done in Kenya and in different parts of Ethiopia, Addiss Ababa, Gondar, Debre Tabore, Bench Shako Zone and Woldia [2,4,13,16,38,39]. Parents’ attitudes, potentially shaped by beliefs about the benefits of HPV vaccination and the severity of cervical cancer, significantly influence their acceptance of the vaccine for their daughters. To foster positive attitudes, communication strategies should highlight the long-term health benefits and feature testimonials from supportive healthcare professionals and parents, specifically targeting those with negative views

Moreover, parents who have positive subjective norms were significantly associated with parents’ acceptance of HPV vaccinations for their daughters. This finding was also supported by other similar studies done in Poland, Colombia, Jeddah, South Africa and Western Nigeria [7,8,25,26,28]. This could be the reason that parents were influenced by peer pressure, health professionals and religious leaders counseling. This was supported by qualitative results, almost all of the participants expressed that counseling is influential for parents’ acceptance of HPV vaccine for their daughters.

Therefore, health care providers in collaboration with other influential stakeholders should give deep counseling service about the importance of HPV vaccine and foster discussions among parents within social networks or parent groups about their decisions regarding HPV vaccination. Encourage positive peer influence by sharing experiences of parents who have chosen to vaccinate their daughter’s targeting for those who have negative subjective norm.

Lastly, safety concern about HPV vaccination was significantly associated with parent’s acceptance of HPV vaccinations for their daughters. This finding was supported with other studies done in Poland, London (UK), United States and Addis Zemen [19,26,32,37]. Parents with lower safety concerns may be more accepting of HPV vaccines due to a better understanding of their safety and effectiveness. Conversely, high safety concerns, potentially stemming from a lack of adequate information, are a major barrier, as highlighted by a Saudi study where 83.8% felt there was insufficient information available [8].

This was also supported by qualitative findings. When asked about community’s safety concern of HPV vaccine towards vaccination of their daughters, one of the key informants expressed that there were false rumors towards HPV vaccine among the community. In depth interview participants think that their daughters will became infertile while vaccinated against HPV infection. In addition, they also think that HPV vaccine was like contraceptive or birth control method and they suspected that it may expose their daughters to risk of sexual behavior.

Therefore, to increase HPV vaccine acceptance among parent’s transparent information about the safety profile of the HPV vaccine based on scientific evidence, address common myths and misconceptions about HPV vaccine safety, communicate the risks of HPV infection and associated diseases compared to the extremely low risks associated with the vaccine and highlight the rigorous safety testing and monitoring processes vaccines undergo before approval by targeting those parents who had high safety concern for HPV vaccine should be important.

Finally, interpretation of this study finding should consider the following limitations: First, the study’s cross-sectional design nature, which limits the ability to establish causal relationships between variables. Second, the reliance on self-reported measures for assessing acceptance and attitudes toward HPV vaccination may lead to response bias. Parents might overestimate their acceptance or knowledge due to social desirability, leading to inaccurate data.

## Conclusions

The result of this study seems promising as a more than two third of women accept to vaccinate their daughters against human papilloma virus though there are still misconceptions, safety and efficacy concerns. Knowledge, attitude, safety concern and subjective norm were factors associated with parent’s acceptance of HPV vaccine for their daughters. The qualitative finding revealed that fear of side effects (false rumors, infertility, perceiving it as contraceptive method), lack of knowledge about HPV infection (exposure to sun (*MECH*), lack of hygiene), lack of responsibility (fear of vaccine shortage, access not fixed) were the barriers whereas, influential stakeholders (healthcare providers, religious leaders, peer pressure), perception of HPV infection (early marriage, unsafe sex(rape), and information, benefit of HPV vaccine (Pre-cancer prevention and to be healthy) and belief about HPV vaccine(HPV vaccine is safe and effective) were facilitators towards parents acceptance of HPV vaccine for their daughters. Hence, to foster cervical cancer prevention efforts, health education should be given in collaboration with other stakeholders to reduce vaccination safety concerns, to increase their level of knowledge and to build positive attitude towards HPV vaccinations of their daughters.

## Supporting information

S1 Data Complete dataset used for the analysis, including socio-demographic variables, parents’ HPV vaccine acceptance, and related factors. (XLSX)
